# Dyadic Prenatal Coparenting Interaction Behaviors Predicting Postpartum Depressive Symptoms during the Transition to Parenthood

**DOI:** 10.3390/ejihpe14060114

**Published:** 2024-06-13

**Authors:** Roi Estlein, Dana Shai

**Affiliations:** 1 School of Social Work, University of Haifa, Haifa 3498838, Israel; 2 School of Psychology, The Academic College of Tel Aviv Yaffo, Tel Aviv-Yafo 6818211, Israel; danamc@mta.ac.il

**Keywords:** postpartum depressive symptoms, prenatal coparenting, transition to parenthood, coparenting

## Abstract

Postpartum depressive symptoms constitute a common yet serious complication of pregnancy and childbirth, but research on its association with coparenting is scarce. Furthermore, although coparenting dynamics start forming prior to the child’s birth, no research has explored dyadic prenatal coparenting dynamics as a predictor of postpartum depressive symptoms. The current study assessed how dyadic prenatal coparenting behaviors predicted postpartum depressive symptoms in first-time parents. We conducted a dyadic mixed-method longitudinal study of 107 expectant couples with data collected prenatally, and at 3, 6, and 24 months post-birth. The results indicated that prenatal coparenting dyadic synchrony predicted low levels of depressive symptoms among first-time fathers 3 and 6 months after the birth, and a prenatal coparenting dynamic of dyadic negative escalation predicted high levels of depressive symptoms among first-time mothers at 3 and 24 months postpartum. The theoretical and practical implications are discussed.

## 1. Introduction

Postpartum depression (PPD) is a serious yet relatively common pathology that can have deleterious implications for the individual, as well as for all family members and family relations, including the developing parent–infant bond [1,2]. Among the predictors of postpartum depressive symptoms, research has highlighted genetic, psychiatric, socioeconomic, and health-related factors, as well as adverse life events and characteristics of social support (see [3,4]). A significant gap in this literature is its overlooking of the potential of the coparenting relationship to constitute a contributing factor to postpartum depressive symptoms; despite constituting one of the most significant and immediate relational environments for parents after the birth of their infant, the coparenting relationship has scarcely been explored in this context.

Coparenting, which concerns the aspects of parenting within the marital relationship (see [5,6]), is central to the daily experience of mothers and fathers. However, the association between coparenting and symptoms of postnatal depression is not fully understood. Understanding the relationship between coparenting and postpartum depressive symptoms is particularly important among first-time parents, because this is the time when partners are co-constructing their shared parenting for the first time and may encounter novel challenges they need to address together. Considering the significance of intimate partners in offering support and an emotional sense of security during the challenging period of transitioning to parenthood [7], it is essential to fill this gap in the literature and to examine the patterns of dyadic coparenting interaction dynamics as a potential indicator of the likelihood of experiencing depressive symptoms after the birth.

From a family developmental perspective [8,9], the transition to parenthood, like all family transitions, evolves gradually over time, so coparenting is first formed when partners prepare for parenting, prior to the birth. In this sense, changes occurring after the birth can already be identified during pregnancy. Indeed, although previous findings did highlight associations between expectant parents’ expectations about their coparenting relationships prior to the birth and coparenting adjustment after the birth [10,11], research on these associations is rare, calling for further exploration of the role of prenatal coparenting in postpartum family processes, such as postpartum depression.

In light of these gaps and limitations in the literature, the current study’s aim is to explore the association between dyadic patterns of prenatal coparenting interaction and postpartum depressive symptoms in first-time mothers and fathers. With the increased stress brought on by the addition of an infant [12], the nature of the coparenting interaction can add or alleviate stress and contribute to the emotional experiences of new mothers and fathers. Indeed, recent initial findings provide support for this assertion, pointing to associations between the individual communication behaviors of each partner (e.g., attempts to avoid interaction or offering positive suggestions to their partner) during prenatal coparenting interaction and postpartum depressive symptoms [13]. Family systems theories [14], however, suggest that partners are interdependent, calling for employing an interpersonal approach to explore the dyadic patterns of partners’ interaction and their potential outcomes. Thus, we draw on a family systems perspective, on evidence that suggest that partners already start to form a coparenting relationship during pregnancy, e.g., [13,15], and on family developmental perspectives that suggest that parents’ experience post-birth can be predicted by their pre-birth functioning [16], we explore the relationship between expectant parents’ dyadic coparenting communication behaviors at the prenatal stage and their postnatal emotional experience in the form of postpartum depressive symptoms. Specifically, we test the hypothesis that prenatal coparenting dyadic interaction patterns will predict postpartum depressive symptoms among first-time mothers and fathers at 3, 6, and 24 months post-birth (whereas for many parents, PPD lasts for several weeks or months, for others, it can continue beyond the first year after the birth, and even several years postpartum [17,18]).

## 2. Materials and Methods

### 2.1. Participants

This study was part of a larger longitudinal research project on the transition to parenthood conducted with 107 Israeli families. It included first-time heterosexual couples in the third trimester of pregnancy to 24 months post-birth. The inclusion criteria included (1) two-parent families expecting their first child and (2) singleton pregnancy, and the exclusion criteria included (1) significant pregnancy complications, (2) significant physical illness, especially cardiovascular diseases, (3) psychiatric disorders, and (4) the use of substantial medication or other substance (including heavy smoking) for full details and a description of the recruitment process, see [13]. The sample size was a priori determined by a power analysis using GPower* in order to reach 95% power to detect medium 0.15-sized effects. Families included co-habiting heterosexual couples who were expecting their first child (*M* = 29.7 weeks pregnant, SD = 2.55 weeks). The study was approved by the local Institutional Review Board (IRB; approval number: 201402).

### 2.2. Procedure

The current study included four sampling time points: prenatal (T1), three months (T2), six months (T3), and 24 months postpartum (T4). At T1, partners arrived at the laboratory and were video-recorded during the Inconsolable Doll Task (IDT) procedure [19,20]. In this validated procedure [19], a doll with a similar physical appearance, size, and weight (2.95 kg) to a baby was used to generate the semblance of a real infant. The doll (RealCare Baby II-Plus; Realityworks, Eau Claire, WI, USA) was equipped with a range of lifelike crying sounds crafted to evoke genuine caregiving and nurturing instincts. In the procedure, a research assistant showed the participants how to nurture the infant, and how it responded to parental care. For a full description of the IDT procedure, and its validation, see [19].

The procedure included one parent staying with the “baby” (counterbalanced), while their partner waited in a separate room. The doll was discretely programmed to be non-responsive to any parental care, creating a situation where it starts and stops crying regardless of the parent’s actions. After 5 min, the parent who was waiting in the other room was asked to join and assist the first parent in calming and soothing the “baby” for 5 additional minutes while the doll remained non-responsive. The simulation of a realistic yet stressful situation created conditions for the partners to become engaged in a prenatal coparenting interaction where their communication behaviors could be more or less collaborative or conflictual.

During the prenatal visit (T1), and during all four time points, participants completed self-report measures with regard to depressive symptoms (at the prenatal time point, they reported their current depressive symptoms, and at the next three time points after birth, they reported depressive symptoms that directly referred to their experience as parents).

### 2.3. Measures

**Prenatal Coparenting Interaction.** To evaluate coparenting dyadic interaction behaviors during the IDT procedure, we used the Interactional Dimensions Coding System IDCS; [21]. The dyadic variables in this coding system highlight interactional dimensions that include the dyadic behavioral attempts of the participating expectant parents to create and demonstrate communication exhibiting support or conflict. To make sure that the evaluations referred to coparenting dynamics rather than marital ones, we coded only communication messages pertaining to the care of the doll (e.g., expectant father: “Your child eats a lot!”; expectant mother: “My baby?! And what, when he is calm, he is your baby?”). The interactional dimension of the coding system includes four scales: (1) negative escalation, reflecting the back-and-forth reciprocated process where both partners rely on destructive communication and negative affect; (2) dominance, evaluating the asymmetries in control between the partners during the task; (3) editing, which assesses the extent of asymmetries in partners’ attempts to stop and avoid negative escalation; and (4) interactional synchrony, which evaluates the degree to which partners co-construct and generate a sense of harmony and coordination during the procedure. The score for each scale was given on a 9-point Likert-type scale ranging from very low (1) to very high (9). Three trained coders (graduate students) coded the interactions. For further details on the coding process, see [13]. The intraclass correlations (ICCs) were 0.89 for negative escalation; 0.93 for dominance; 0.73 for editing; and 0.85 for synchrony.

**Depressive Symptoms.** We used Cox et al.’s [22] Edinburgh Postnatal Depression Scale (EPDS) to assess participants’ postpartum depressive symptoms. Ranging from 0 to 3, the EPDS consists of 10 statements pertaining to parents’ feelings during the last seven days (e.g., “I have been able to laugh and see the funny side of things”, with 0 representing “as much as I ever could” and 3 representing “not at all”). A participant’s score is the sum of their responses and can range from 0 to 30, with higher scores displaying more intense depressive symptoms (*M* = 5.19, SD = 3.33, *α* = 0.85 for fathers; *M* = 5.95, SD = 4.07, *α* = 0.89 for mothers). To conduct an assessment of the participants’ depressive symptoms pre-birth as a baseline, we employed the depression sub-scale from the Brief Symptom Inventory (BSI) [23]. This scale ranges from 1 (not at all) to 5 (all the time) and reflects participants’ depressive symptoms during the past week (e.g., “feeling no interest in things”) (*M* = 3.69, SD = 3.32, *α* = 0.79 for fathers; *M* = 4.45, SD = 3.47, *α* = 0.82 for mothers).

The **control variables** included gender, age, and income, as well as prenatal expectations of coparenting relations, perceived postnatal coparenting relations (CRS) [24], and perceived couple relationship quality (CSI) [25].

### 2.4. Data Analysis

We started with a preliminary analysis of paired-sample *t*-tests to identify differences between male and female participants, and bivariate correlations to document the nature of the associations between the studied variables. We then conducted multilevel modeling for testing our hypothesis. For our preliminary analysis, we used SPSS 28.0, and for our substantive analysis, we used HLM 8.2. Due to the small number of missing data in our study, we used Full Information Maximum Likelihood (FIML) estimation, which uses all available information from the observed responses in the data; this way, cases with missing scores are not “deleted” or excluded from the analysis, but rather, incomplete cases’ observed scores are estimated by their likelihood function, which maximizes accuracy and statistical power [26].

## 3. Results

### 3.1. Preliminary Analysis

We first wanted to explore any potential differences between males and females in our study. Paired-sample *t*-tests indicated significant differences between first-time fathers’ and mothers’ postpartum depressive symptoms at 6 months post-birth (*t* (95) = −2.45, *p* = 0.01), with fathers reporting lower levels of postpartum depressive symptoms (*M* = 4.49, *SD* = 3.42) than mothers (*M* = 5.88, *SD* = 4.28), and between mothers’ and fathers’ prenatal communication skills (*t* (95) = −1.33, *p* < 0.001), such that mothers (*M* = 4.88, *SD* = 1.65) scored higher than fathers (*M* = 3.95, *SD* = 1.52) in the IDT. No significant differences were found between mothers and fathers in all other aspects of their prenatal coparenting interaction behaviors in postpartum depressive symptoms either 3 or 24 months after the birth.

Next, we conducted a bivariate correlation analysis among the variables in the prenatal stage and at the three postnatal time points (see Table 1). The results indicated that for fathers, postpartum depressive symptoms at 3 and 6 months were negatively associated with prenatal observed interactional synchrony (*r* = −0.24, *p* < 0.05 and *r* = −0.27, *p* < 0.05, respectively), and for mothers, postpartum depressive symptoms at 3 and 24 months were positively associated with prenatal observed negative escalation (*r* = 0.25, *p* = 0.05 and *r* = 0.26, *p* < 0.05, respectively). In terms of the control variables, age, income, and prenatal coparenting expectations were not significantly associated with postpartum depressive symptoms, whereas gender (*r* = 0.21, *p* < 0.05), perceived relationship quality (*r* = −0.13, *p* < 0.05), and perceived postnatal coparenting relationship (*r* = −0.41, *p* < 0.01) were significantly associated with postpartum depressive symptoms.

### 3.2. Substantive Analyses and Test of Hypothesis

Our main goal in this study was to explore the potential contribution of dyadic prenatal coparenting interaction behaviors to postpartum depressive symptoms among first-time mothers and fathers. After assessing the bivariate correlations of this association, we further examined whether the observed dyadic prenatal coparenting interaction behaviors predicted postpartum depressive symptoms above and beyond other potential associated variables, including partners’ prenatal expectations about coparenting and postnatal coparenting quality, prenatal depressive symptoms, and relationship quality. We used the Hierarchical Linear Model (HLM8.2) software, which is designed to both accommodate nested data [27] and analyze non-independent dyadic data [28]. Importantly, because the predictor variables in this study (i.e., observed prenatal coparenting dynamics) were only assessed one time, namely, prenatally, using models for repeated measures was not appropriate. Moreover, because research on postpartum depression points to variability in the onset, intensity, and course of development of postpartum depressive symptoms across individuals [29], we wanted to explore and highlight the idiosyncratic relations independently between the variables in our study at each time point after the birth. For these two reasons, we produced separate independent models for each time point (see also [19,30,31]). Due to the dyadic nature of our data, however, we did analyze them using two-intercept models, where both partners were included, to account for couple interdependence and for any dyadic variability, and to allow for the consideration of estimating effects for male and female participants both simultaneously and separately (see [32,33]).

Thus, we constructed each model to include one predictor (i.e., prenatal dyadic synchrony and negative escalation) and an outcome variable (i.e., postpartum depressive symptoms at 3, 6, and 24 months after birth). Within the model, gender was evaluated as a moderator, and dyads were distinguished by gender (men = −1, women = 1). Each model also included the covariates (i.e., prenatal coparenting expectations, postnatal coparenting quality, prenatal depressive symptoms, and couple relationship quality), and the interaction terms for gender with each predictor to identify potential differences between male and female partners.

The results are presented in Table 2, indicating significant main effects for prenatal depressive symptoms at 3 (but not at 6 or 24) months post-birth, relationship quality at all three time points, postnatal coparenting perceptions at 3 and 6 (but not 24) months post-birth, gender at 6 (but not 3 or 24) months post-birth, and both couple synchrony and negative escalation at all three time points. The interaction terms suggested that interactions for dyadic synchrony were significant at 3 and 6 months post-birth, and for negative escalation and gender at 3 and 24 months post-birth, suggesting differences between male and female participants in these variables; thus, using a two-intercept approach, we tested the interactions to calculate the simple slopes for male and female participants. The results showed that prenatal dyadic synchrony predicted postpartum depressive symptoms for fathers at 3 and 6 months post-birth, but not for mothers. Prenatal negative escalation predicted postpartum depressive symptoms for mothers at 3 and 24 months post-birth, but not for fathers (see Table 2 for details).

## 4. Discussion

The main goal of this study was to examine dyadic prenatal coparenting interaction behaviors as a predictor of postpartum depressive symptoms among first-time mothers and fathers throughout the transition to parenthood. Our findings highlighted two aspects of dyadic prenatal coparenting behaviors as predicting depressive symptoms post-birth, namely, negative escalation and dyadic synchrony. More specifically, dyadic synchrony observed in coparenting interaction pre-birth was predictive of low levels of postpartum depressive symptoms among first-time fathers, and negative escalation observed in coparenting interaction pre-birth was predictive of high levels of postpartum depressive symptoms among first-time mothers. These results remained even when controlling for prenatal depressive symptoms, prenatal coparenting expectations, postnatal coparenting perceptions, and prenatal couple relationship quality, suggesting that they explain unique variance in postpartum depressive symptoms above and beyond these reports.

This study drew on recent findings that revealed actor and partner effects of individual prenatal coparenting behaviors of first-time parents on postpartum depressive symptoms [13], and extended them by exploring associations between expectant parents’ dyadic interaction patterns before birth and depressive symptoms after birth. This goal was also driven by the understanding that, despite the central role of the emerging coparenting relationship in first-time parents’ daily adjustment and its development already beginning during pregnancy, the association between prenatal coparenting dynamics and postpartum depressive symptoms is extremely understudied. Our results shed light on this association, which is important for understanding the experiences of first-time parents during the transition to parenthood over time. More specifically, these findings highlight the following: first, the role dyadic aspects of prenatal coparenting behavior play in emotional processes and psychological outcomes during the transition to parenthood; second, how positive and negative dyadic aspects of coparenting are both important in predicting psychological outcomes for parents; and finally, the different effects positive and negative dyadic prenatal coparenting behaviors have on mothers and fathers. Importantly, these results should be interpreted considering the characteristics and the context of the sample used in the current study—namely, heterosexual, Israeli, low-risk couples. Nevertheless, these findings highlight the associations between prenatal dyadic factors and postnatal psychological outcomes and the need to look further into these associations, and thus constitute an important novel step toward advancing our understanding of the unfolding experience of the transition to parenthood for first-time parents.

### 4.1. Dyadic Aspects of Prenatal Coparenting Behavior and Depressive Symptoms Post-Birth

First, our study found longitudinal effects of dyadic prenatal coparenting behavior on postpartum depressive symptoms. This finding adds to the growing body of research exploring associations between pre- and post-birth coparenting processes; however, whereas most of that research has provided important information about associations between prenatal and postnatal specific coparenting dynamics, e.g., [10,15], we were able to document how dyadic prenatal coparenting dynamics have consequences for the psychological experience of first-time parents and, specifically, for postpartum depressive symptoms, a highly common mental burden for many parents.

This finding suggests not only that the coparenting relationship already starts to form during pregnancy, but also that aspects of the emerging coparenting relationship predict important post-birth outcomes. This finding contributes significantly to the literature on postpartum depression, which mostly highlights genetic, socioeconomic, and health-related factors, as well as characteristics of prenatal couple relationships and postpartum coparenting as associated with postpartum depressive symptoms [34,35,36,37,38,39,40].

### 4.2. Positive and Negative Dyadic Aspects of Prenatal Coparenting and Depressive Symptoms Post-Birth

Our results also demonstrate the importance of considering negative as well as positive interaction sequences when examining the association between coparenting and family outcomes. Whereas previous studies found that positive prenatal coparenting dynamics, such as solidarity and alliance, were associated with postnatal characteristics [41,42], the current results suggest that both negative escalation and dyadic synchrony in prenatal coparenting interactions are predictive of postpartum outcomes. Moreover, although prior research pointed to both positive [31] and negative (e.g., [43,44]) aspects of postnatal coparenting dynamics as predictive of postpartum depressive symptoms, our findings highlighted the significance of such dyadic patterns in explaining long-term effects as early as during pregnancy.

Importantly, our findings are also in line with recent findings that showed how a positive sense of security in the prenatal couple relationship acts as a protective factor against postpartum depressive symptoms, and high rates of negative hostile communication prenatally are predictive of higher risk for postpartum depressive symptoms among unwed adolescent mothers [45]. The current findings extend these initial findings in at least two ways: first, they highlight specific dyadic coparenting behavioral characteristics (i.e., negative escalation and dyadic synchrony) that underlie the more general senses of security and hostility in the couple’s communication, and may promote such emotions; second, they highlight the effects of these dyadic communication behaviors among non-adolescent married first-time parents, providing evidence for the way prenatal communication predicts postpartum depressive symptoms among non-fragile families [46].

It is worth noting that, surprisingly, although both positive—namely, dyadic synchrony—and negative—namely, negative escalation—interaction behaviors were associated with postpartum depressive symptoms, two other dyadic communication features—dominance and editing—were not. Why may this be the case? First, this finding suggests that not every positive or negative dyadic behavior in couples’ interactions is similarly associated with mental outcomes, and that different interpersonal exchanges seem to differently reflect and shape psychological experiences. This is important because it calls scholars and professionals who work with couples in general, and with first-time parents in particular, to pay close attention to specific interaction behaviors and their implications for each partner and for both. Second, whereas both dyadic synchrony and negative escalation refer to symmetric reciprocal back-and-forth sequences between partners, where they both equally contribute to the shaping and construction of the interaction, both dominance and editing refer to asymmetries between partners in their attempts to control the situation or determine the climate in the interaction. It may be that partners’ shared co-construction of the interaction—whether it is positive or negative—contributes to their experience more significantly than interaction sequences characterized by asymmetry and less reciprocity.

### 4.3. Dyadic Prenatal Coparenting Behaviors and Gender

Finally, our results revealed that whereas postpartum depressive symptoms among fathers were predicted by low levels of prenatal dyadic synchrony, postpartum depressive symptoms among mothers were predicted by high levels of prenatal negative escalation, suggesting that characteristics of dyadic prenatal coparenting dynamics have different effects for first-time fathers and first-time mothers. Tissot et al. [38] highlighted support and conflict as the core components of the coparenting relationship. Our findings seem to correspond with this conceptualization, suggesting that even prior to the birth of the infant, supportive and conflictual coparenting are significant; however, whereas supportive elements (i.e., dyadic synchrony) in prenatal coparenting seem to serve as a potential buffer against paternal postpartum depressive symptoms, conflictual elements (i.e., negative escalation) in prenatal coparenting seem to be linked to maternal postpartum depressive symptoms.

These results may imply that whereas first-time fathers are reactive to supportive coparenting, first-time mothers are more sensitive to conflictual coparenting. Indeed, Solmeyer and Feinberg [37] found that supportive coparenting during early parenthood was predictive of low depressive symptoms for fathers but not for mothers. In addition, previous studies have reported associations between maternal encouragement and father involvement [47] where others pointed at maternal gatekeeping—where mothers limit their partners’ involvement in childcare and childrearing—as a major concern of first-time fathers that may invoke frustration and reduced paternal involvement [48,49]. Olsavsky et al. [50] found that fathers’ perceptions of maternal gatekeeping three months post-birth were associated with lower dyadic adjustment and coparenting closeness at nine months postpartum. Our findings suggest that even prenatally, mutual supportive coparenting advances positive emotion among fathers post-birth, and buffers against paternal depressive symptoms.

In terms of first-time mothers, postnatal inter-parental conflict has been associated with maternal depression [51]. Our results support this finding and add to it by suggesting that coparenting conflict dynamics constitute a warning sign of maternal depression postpartum. Moreover, both maternal adjustment and mothers’ relationship satisfaction have been negatively associated with postnatal couple conflict [52,53]. The current findings highlight the role played by conflictual coparenting in the form of negative escalation even prenatally in predicting postpartum hardships, such as postpartum depressive symptoms, in first-time mothers. Additionally, previous findings show that in heterosexual couples, women, but not men, pay attention to their partners’ contribution to the couple dynamics and are influenced by it [54]. Thus, it could be that undermining conflictual messages made by the male partner are registered in the female partner, leaving an impact on their emotional experience. Additionally, women’s appraisals of negative aspects in the relationship—and negative escalation in coparenting constitutes such an element—are associated with increased daily stress [54] which can manifest as depressive symptoms as well.

### 4.4. Clinical Implications

The findings from the current study have significant clinical implications for working with expectant first-time parents, particularly where partners’ prenatal exchanges about their future shared parenting are characterized by negative escalating sequences of communication. In this sense, first, our findings suggest that it is important that clinicians and therapists pay attention to both negative and positive prenatal communication behaviors as they seem to evoke different responses and implications. Professionals can focus on helping couples who rely on negative communication that escalates during interactions to identify the nature of their exchanges and find ways to create new, less negative patterns of interaction. Additionally, they can help couples spot any positive interpersonal exchange in their interactions to highlight its potential benefits for their shared and individual experiences, and assist them in enhancing its presence in their coparenting discussions. Second, the longitudinal nature of our findings allow us to consider the development of both intervention and prevention programs for working with expectant parents. More specifically, our findings offer clinicians a conceptual framework for understanding and identifying specific interaction behaviors in coparenting (i.e., negative escalation and dyadic synchrony) as risk and protective factors as early as during pregnancy as well as post-birth. Helping first-time parents identify and explore their interaction patterns concerning coparenting both pre- and post-birth can assist them in reclaiming responsibility for their mutual behavior and attempting to co-construct their interactions in more effective ways.

## 5. Conclusions

This study offers valuable insights into the dynamics between prenatal coparenting dynamics and postpartum depressive symptoms in first-time parents. Advancing our understanding of the ways that the emerging coparenting relationship is associated with the mental health and psychological experiences of first-time parents extends both theory and practice by providing significant information that can generate a basis for developing preventive programs and interventions to help professionals who work with first-time parents. Specifically, highlighting both supportive and conflictual aspects of coparenting as early as during pregnancy, and taking into consideration the divergent effects they have on first-time fathers and mothers, clinicians and therapists can assist first-time parents to navigate through the challenges of the transition to parenthood.

It is also important to indicate this study’s strengths and limitations, and directions for future research. The present study has significant strengths, such as its longitudinal design, use of both self-reporting and observations, and dyadic data that enabled the exploration of intricate systems-oriented research inquires and highlighted associations between first-time parents’ dyadic prenatal and postnatal experiences. Importantly, our study contributes to the sparse body of observational research on prenatal coparenting interaction behaviors that are associated with postpartum functioning [55], as well as to the literature on the transition to parenthood, with evidence of the role dyadic processes have during this developing transition. This study also has a few limitations. First, because our sample included only low-risk community-based heterosexual couples, the generalizability of our results may be somewhat limited both in terms of higher-risk couples, who may display higher prevalence rates of postpartum depressive symptoms [56], and in terms of non-heterosexual and adoptive parents, whose dyadic coparenting dynamics may or may not be similar to heterosexual first-time parent couples, and thus may result in different associations. Second, despite our longitudinal design, following first-time mothers and fathers along more or less spaced out time points can yield further information on how the psychological and mental health experiences of first-time parents unfold, and their association with the developing coparenting relationship.

Considering the findings, the strengths, and the limitations of this study, future research should look into the associations between prenatal coparenting dyadic interaction features and postpartum depressive symptoms among first-time parents in more diverse family settings. More specifically, researchers should explore the associations studied here in clinical samples (e.g., expectant first-time parents with observed depression), as well in non-heterosexual and non-biological parent couples, who may exhibit distinct patterns of prenatal and postnatal coparenting dynamics that may or may not be associated differently with postpartum depressive symptoms. In addition, research can extend our understanding of the interplay between prenatal coparenting interaction dynamics and postpartum depressive symptoms over time by following parents at different—closer or farther—time points throughout the transition to parenthood.

## Figures and Tables

**Table 1:** Bivariate correlations among the studied variables over three time points.

	1	2	3	4	5	6	7	8	9	10
1. IDCS Negative Escalation	-									
2. IDCS Dominance	−0.02	-								
3. IDCS Editing	0.34 **	0.34 **	-							
4. IDCS Synchrony	−0.20	−0.38 **	−0.32 **	-						
5. Paternal EPDS at 3 M	−0.02	0.14	0.01	−0.24 *	-					
6. Paternal EPDS at 6 M	−0.08	0.18	0.04	−0.27 *	0.70 **	-				
7. Paternal EPDS at 24 M	0.04	0.15	0.19	−0.21	0.45 **	0.44 **	-			
8. Maternal EPDS at 3 M	0.25 *	0.05	0.01	0.10	0.18	0.11	0.17	-		
9. Maternal EPDS at 6 M	0.13	−0.02	−0.06	0.03	0.09	0.09	0.14	0.59 **	-	
10. Maternal EPDS at 24 M	0.26 *	−0.02	0.06	0.07	0.03	0.01	0.11	0.34 **	0.49 **	-

* *p* < 0.05 ** *p* < 0.01.

**Table 2:** Dyadic synchrony and negative escalation predicting PPD at 3, 6, and 24 months.

	3 M Postpartum				6 M Postpartum				24 M Postpartum			
	Dyadic Synchrony		Negative Escalation		Dyadic Synchrony		Negative Escalation		Dyadic Synchrony		Negative Escalation	
	*β*	*T*-Ratio	*β*	*T*-Ratio	*β*	*T*-Ratio	*β*	*T*-Ratio	*β*	*T*-Ratio	*β*	*T*-Ratio
Intercept	2.55 ***	6.79	2.55 ***	6.87	2.66 ***	6.99	2.71 ***	6.97	2.85 ***	5.48	2.84 ***	5.48
Slope for covariates												
Gender	0.10	0.30	0.06	0.27	0.27 *	2.53	0.27 *	2.59	0.09	0.39	0.09	0.31
Prenatal coparenting expectations (CRS)	0.06	0.61	0.07	0.79	0.24	0.26	0.43	0.51	0.58	1.49	0.32	1.25
Prenatal depressive symptoms (BSI)	0.31 *	2.21	0.31 *	2.21	0.20	1.36	0.20	1.19	0.15	0.85	0.09	0.51
Relationship quality (CSI)	−0.17 *	−2.09	−0.09 *	−1.95	−0.13 *	−1.25	−0.17 *	−1.29	−0.13 *	−1.15	−0.12 *	−1.09
Postnatal coparenting perceptions	−0.21 *	−1.90	−1.04 *	−1.27	−0.69 **	−2.74	−0.46 *	−1.59	−0.39	−0.86	−0.21	1.07
Slope for predictors												
Synchrony	−17 *	−1.95			−0.46 *	−2.55			−0.30 *	−1.10		
Negative escalation			0.29 *	2.57			0.23 *	2.39			0.42 **	2.10
Interactions for predictors												
Synchrony × gender	−0.15 *	−2.09			−0.23 **	−1.75			−0.19	−1.05		
Negative escalation × gender			0.23 *	1.96			0.11	0.73			0.29 *	2.59

* *p* < 0.05 ** *p* < 0.01 *** *p* < 0.001.

## Data Availability

The datasets generated and analyzed during the current study are available from the second author on reasonable request.
